# Using the Observational Medical Outcomes Partnership Common Data Model for a multi-registry intensive care unit benchmarking federated analysis: lessons learned

**DOI:** 10.1093/jamiaopen/ooaf052

**Published:** 2025-07-22

**Authors:** Aasiyah Rashan, Daniel P Püttmann, Nicolette F de Keizer, Dave A Dongelmans, Ronald Cornet, Otavio Ranzani, Wangari Waweru-Siika, Matthew Smith, Steve Harris, Abi Beane, Ferishta Bakhshi-Raiez, Collaboration for Research, Implementation and Training in Critical Care—Asia and Africa Investigators, and the Dutch National Intensive Care Registry, Roya Afzali, Noorullah Ahmadzai, Mirwais Azizi, Nasibullah Barukzai, Maryam Barukzay, Naqibullah Danish, Maliha Farooq, Maryam Shamal Ghalib, Owais Urhman Ghalib, Rahim Mazloomyar, Shoaib Mirzada, Meher Negar, Bahar Nadim, Abdul Majid Rahimi, Muhammad Dawood Safi, Muhammad Hamid Rahimi Safi, Guldad Khan Saifi, Ahmad Zakariya Shinwary, Hiranmoy Dutta, Enshad Ekramullah, Aniruddha Ghose, Md Hassanuzzaman, Muna Islam, Mahabubul Alam Khondokar, Md Abdur Rahim, Md Harun Or Rashid, Md Abdus Sattar, Abdullah Abu Sayeed, Sarkar Shoman, Md Rezaul Hoque Tipu, Rabiul Alam Md Erfan Uddin, Mohammed Jashim Uddin, A S M Zahed, Menbeu Sultan, John Amuasi, Joe Bonney, Moses Siaw Frimpong, Mohd Shahnaz Hasan, Mohd Basri Mat Nor, Mohd Zulfakar Mazlan, Isha Amatya, Diptesh Aryal, Basanta Gauli, Praveen Giri, Kishor Khanal, Sushil Khanal, Sabin Koirala, Sanjay Lakhey, Subekshya Luitel, Hem Raj Paneru, Sushila Paudel, Lalit Rajbanshi, Sangina Ranjit, Yam Roka, Pramesh Sundar Shrestha, Raju Shrestha, Pradeep Tiwari, Wangari Waweru-Siika, Madiha Hashmi, Eva Hanciles, Luigi Pisani, Dave Thomson , Martha Alupo, Adam Hewitt Smith, Dennis Kakaire, Herbert Kiwalya, Joseph Kiwanuka, Arthur Kwizera, Joshua Muhanguzi, Cornelius Sendagire, Udara Attanayake, Abi Beane, Sri Darshana, Arjen M Dondorp, Layoni Dullewe, Nilmini P Dullewe, Kaumali Gimhani, Judy Ann Gitahi, Rashan Haniffa, Pramodya Ishani, Chamira Kodippily, Issrah Jawad, Shiekh Mohiuddin, Himasha Muvindi, Upule Pabasara, Luigi Pisani, Dilanthi Priyadarshani, Disna Pujika, Aasiyah Rashan, Sumayyah Rashan, Thalha Rashan, Shoba Sathasivam, Timo Tolppa, Shara Udayanga

**Affiliations:** Institute of Health Informatics, University College London, London WC1E 6BT, United Kingdom; Department of Medical Informatics, Amsterdam Public Health Institute, Amsterdam UMC, University of Amsterdam, Amsterdam 1105 AZ, The Netherlands; National Intensive Care Evaluation (NICE) Foundation, Amsterdam 1105 AZ, The Netherlands; Quality of Care, Amsterdam Public Health Institute, Amsterdam 1105 AZ, The Netherlands; Department of Medical Informatics, Amsterdam Public Health Institute, Amsterdam UMC, University of Amsterdam, Amsterdam 1105 AZ, The Netherlands; National Intensive Care Evaluation (NICE) Foundation, Amsterdam 1105 AZ, The Netherlands; Quality of Care, Amsterdam Public Health Institute, Amsterdam 1105 AZ, The Netherlands; National Intensive Care Evaluation (NICE) Foundation, Amsterdam 1105 AZ, The Netherlands; Department of Intensive Care, Amsterdam University Medical Centers Location Academic Medical Center, Amsterdam 1105 AZ, The Netherlands; Department of Medical Informatics, Amsterdam Public Health Institute, Amsterdam UMC, University of Amsterdam, Amsterdam 1105 AZ, The Netherlands; Digital Health, Amsterdam Public Health Institute, Amsterdam 1105 AZ, The Netherlands; Barcelona Institute for Global Health, ISGlobal, 08036 Barcelona, Spain; DataHealth Lab, Institut de Recerca Sant Pau (IR SANT PAU), 08041 Barcelona, Spain; Department of Anaesthesia, Aga Khan University, Nairobi 30270-00100, Kenya; Department of Medical Statistics, London School of Hygiene and Tropical Medicine, London WC1E 7HT, United Kingdom; Institute of Health Informatics, University College London, London WC1E 6BT, United Kingdom; Pandemic Science Hub, Institute of Regeneration and Repair, University of Edinburgh, Edinburgh EH16 4UU, United Kingdom; Department of Medical Informatics, Amsterdam Public Health Institute, Amsterdam UMC, University of Amsterdam, Amsterdam 1105 AZ, The Netherlands; National Intensive Care Evaluation (NICE) Foundation, Amsterdam 1105 AZ, The Netherlands; Quality of Care, Amsterdam Public Health Institute, Amsterdam 1105 AZ, The Netherlands

**Keywords:** federated analysis, global health, data standardization, OMOP CDM, APACHE II, registries, critical care

## Abstract

**Objective:**

Federated analysis is a method that allows data analysis to be performed on similar datasets without exchanging any data, thus facilitating international research collaboration while adhering to strict privacy laws. This study aimed to evaluate the feasibility of using federated analysis to benchmark mortality in 2 critical care quality registry databases converted to the Observational Medical Outcomes Partnership (OMOP) Common Data Model (CDM), describing challenges to and recommendations for performing federated analysis on data transformed to OMOP CDM.

**Materials and Methods:**

To identify as many challenges as possible and to be able to complete the benchmarking phase, a 2-step approach was taken during implementation. The first step was a naive implementation to allow challenges to surface naturally; the second step was developing solutions for the encountered challenges. Expected patient mortality risk was calculated by applying the Acute Physiology and Chronic Health Evaluation II (APACHE II) model to data from OMOP CDM databases containing adult ICU encounters between July 1, 2019 and December 31, 2022. An analysis script was developed to calculate comparable, registry level standardized mortality ratios. Challenges were recorded and categorized into predefined categories: “data preparation,” “data analysis plan,” and “data interpretation.” Challenges specific to the OMOP CDM were further categorized using published steps from an existing generic harmonization process.

**Results:**

A total of 7 challenges were identified, 4 of which were related to data preparation, 1 to data analysis, and 1 to data interpretation. Out of all 7 challenges, 4 stemmed from decisions made during the implementation of OMOP CDM. Several recommended solutions were distilled from the naive approach.

**Discussion:**

Federated analysis facilitated by a CDM is a feasible option for critical care quality registries. However, future analysis is influenced by decisions made during the CDM implementation process. Thus, prior publication of data dictionaries and the use of metadata to communicate data handling and data source classification during CDM implementation will improve the efficiency and accuracy of subsequent analysis.

## Objective

International research collaboration is a significant opportunity for critical care quality registries (henceforth referred to as quality registries for brevity). These registries aim to facilitate the improvement of critical care services through benchmarking, facilitating registry embedded research, and providing data for audit and feedback. Benchmarking is the process of comparing risk adjusted outcomes over a specific period of time, across different peers or sites, and among patient populations to identify opportunities for improvement, learn from best performers, and enhance quality of care.^1–4^ The recent COVID-19 pandemic prompted efforts to expand existing quality registry databases internationally. In response, quality registries demonstrated their potential to generate data for infectious disease-specific surveillance, service forecasting, and clinical research.^5^^,^^6^

The COVID-19 pandemic also highlighted limitations in the ability of quality registries to collaborate for research internationally.^7–9^ Quality registry datasets are rarely aligned with each other, as they are typically designed to meet the specific needs and capabilities of individual health systems. For example, a universal feature of quality registries is the inclusion of a specific severity of illness score calculated with prognostic models. These models are developed to enable case-mix adjustment, allowing for equitable comparison of health outcomes across a diverse patient population within these registries. However, the implementation of prognostic models to calculate severity of illness scores may differ between quality registries and geographic regions. Until recently, efforts to standardize datasets and prognostic score calculation internationally have been hampered by concerns about data availability and heterogeneity of the global critical care population and care processes.^10^

Quality registry datasets require substantial transformation, if the datasets are to be harmonized and made suitable for international collaborative research. Traditionally, this involved transferring data to a central location for cleaning and transformation prior to its analysis. However, such processes are inefficient and increasingly prohibitive to the international community. This is particularly true for countries where cooperation is impeded by strict data sharing regulations.^11–13^ An increasingly proposed solution is federated analysis, which involves conducting analysis on multiple datasets using shared analysis scripts, without the need to physically relocate the data to a central repository. Casaletto et al. call it “bringing the code to the data,” rather than the reverse.^14^

For federated analysis to be successful, datasets need to align in data models, data elements, and terminology systems. Common data models (CDMs) have sought to provide such standardization and enable data to be analyzed without inspecting source data. Multiple CDMs have been proposed based on their suitability for heterogeneous patient populations and their applicability to clinical documentation. Examples of CDMs include those which standardize trial data^15^ and longitudinal data for primary care.^16^ Such CDMs further utilize existing terminology systems like Systematized Nomenclature of Medicine Clinical Terms (SNOMED CT) and Logical Observation Identifiers Names and Codes (LOINC) to reduce ambiguity over data elements and their values.^17–21^

Quality registries are increasingly adopting the Observational Medical Outcomes Partnership (OMOP) Common Data Model (CDM) as a framework for standardizing data.^22–24^ Developed by the Observational Health Data Sciences and Informatics (OHDSI) collaborative, this CDM offers a standardized table structure for a wide variety of observational data and additional open access tools for transforming the data and conducting federated analyses. However, to our knowledge, no studies have been published on the feasibility of doing federated analysis on critical care registry data. Therefore, this study aims to evaluate the feasibility of federated analysis to benchmark mortality outcomes on 2 quality registry databases converted to the OMOP CDM and to describe challenges and recommendations for performing a federated analysis on data transformed to the OMOP CDM.

## Materials and methods

### Data sources

This study used the OMOP CDM databases of 2 quality registries: the National Intensive Care Evaluation (NICE),^1^ and the Collaboration for Research, Implementation and Training in Critical Care Asia and Africa (CCAA).^2^ NICE includes data from Dutch Intensive Care Units (ICUs), and CCAA is a network of 17 quality registries operational in countries in Africa and Asia. These quality registries are members of the LOGIC international critical care benchmarking consortium^3^ and have both independently applied Extract, Transform, and Load (ETL) processes^25^ to generate their OMOP CDM databases.

The OMOP CDM has a relational table structure centered around a “person,” for example, an ICU patient. This person has a visit occurrence and visit detail, which map to a hospital encounter and ICU encounter respectively. Linked to the person and their visits are measurements, condition occurrences, and procedure occurrences, examples being blood pressure, diabetes, and dialysis, respectively. Any variable that does not fit into the previous 3 categories is mapped to observation, such as a length of stay.^26^

The NICE registry was established in 1996 and receives data from all Dutch adult ICUs since 2016.^1^ Data are gathered from existing electronic health records (EHRs), where it is manually and automatically validated by trained intensivists, ICU-nurses, or data managers, and uploaded monthly to the NICE database. Data include demographics, comorbidities, reasons for admission, highest and lowest laboratory results and physiology in the first 24 hours of ICU admission, organ support information, and patient-level ICU and hospital outcomes such as length of stay and mortality.^27^ Consequently, the following OMOP tables were filled in the NICE OMOP database: person, visit occurrence, visit detail, condition occurrence, procedure occurrence, measurement, observation, observation period, and death in ICU.^26^ NICE was standardized using OMOP CDM version 5.3, and vocabulary version v20220510.^22^ The OHDSI Achilles tool and the OHDSI Data Quality Dashboard, which run data quality checks on OMOP databases using a harmonized framework, was implemented and the database passed the checks.^22^^,^^28^^,^^29^

CCAA was formally established in 2020, with quality registries collecting data prospectively from 2019.^2^ Data are manually gathered daily from paper or electronic records by trained data collectors overseen by trained intensivists and received through a common data platform. The dataset includes information on patient demographics, comorbidities,^30^ reasons for admission (entered using SNOMED CT terms),^31^ highest or lowest laboratory and point of care tests, and physiology and organ support in the first 24 hours of ICU admission along with ICU and hospital outcome.^2^ The CCAA OMOP database contains all OMOP CDM tables used in the NICE OMOP database described above, with the addition of the care site and drug exposure tables.^26^ CCAA was standardized using OMOP CDM version 5.4, and vocabulary version v20220602. The OHDSI Data Quality Dashboard was implemented, and the database passed the checks.^29^

### Federated analysis for multi-registry benchmarking

#### Severity of illness score

The standardized mortality ratio (SMR) is a commonly used measure for benchmarking case-mix adjusted outcomes in patient populations. It is the ratio of observed deaths to expected deaths over a time frame and is an indicator of the quality of care. A ratio of one means the quality of care is as expected, a higher ratio indicates a possible deterioration, and a lower ratio indicates a possible improvement.

To calculate the SMR, the observed mortality at ICU discharge in each ICU was divided by its expected mortality, which is the mean of predicted mortality risks in all patients multiplied by the number of patients. The risk of mortality was calculated using the Acute Physiology and Chronic Health Evaluation II (APACHE II) model.^32^ Although APACHE II was developed to predict hospital mortality, ICU mortality was chosen as the outcome since hospital mortality is not routinely available in the CCAA dataset and external validations have suggested that APACHE II can perform sufficiently on this alternative outcome.^33^ Although superseded by APACHE IV, APACHE II is validated internationally and is still widely used to describe population severity of illness and outcomes for critical care research. Moreover, all its component variables are routinely collected by both NICE and CCAA.^33–35^

#### Population selection

For NICE, all data were included. For CCAA, 12 collaborating quality registries which had previously agreed on transforming their data to the OMOP CDM format were included. From both sources, all patients admitted to adult ICUs between July 1, 2019 and December 31, 2022 were included. Following APACHE II exclusion criteria, patients were excluded if they were less than 17 years of age or had a diagnosis of burns.^32^ Patients without an APACHE II reason for admission recorded were also excluded. If patients had multiple ICU admissions during a hospital admission, only data from their first admission were used.^32^

#### Analysis

For each data source, demographic information, the APACHE II score, and patient ICU outcomes were reported as N (%) or Median (IQR). The availability and distribution of each component variable for APACHE II was reported for both databases. SMRs were calculated per ICU. To visualize the variation in SMRs among different ICUs, so-called funnel plots were used. In such a plot, each ICU is represented as a dot. The value on the x-axis indicates the expected number of deaths for the respective ICU, while the value on the y-axis represents the SMR. The horizontal line represents the average SMR, which has a value of 1. The curved lines represent the corresponding 95% control limits (CL), emphasizing that there is no single “normal” SMR value. Instead, a range of values is considered normal depending on the expected number of deaths.^36^

In the extraction scripts, Structured Query Language (SQL) was used to select patients meeting eligibility and inclusion criteria. The OHDSI R package SQLRender version 1.16.1 was used to translate SQL queries between SQL dialects.^37^ R software version 4.1 was used for data analyses.^38^ The federated analysis was performed by running the same analysis scripts separately on both data sources. Analysis scripts were developed collaboratively and stored on GitHub and Figshare, see Data availability section.

#### Two-step approach

To identify as many challenges as possible, and to be able to perform the federated analyses, a 2-step approach was taken during implementation. For the naive approach, the study group agreed on the analysis plan above. Author A.R. then developed an analysis script for the CCAA database, and shared it with author D.P., who attempted to apply it on the NICE database. Challenges identified during this first step were recorded and categorized, see details in the section below. In the second step, the study group met to discuss challenges encountered during the first step. They agreed on revisions to the initial analysis plan and identified solutions, which A.R. and D.P. implemented to complete the federated analysis. The solutions are described in more detail in the “Results” section.

#### Categorization of challenges

Challenges identified during step 1 were recorded, and discussed by the authors A.R., D.P., N.K., D.D., R.C., A.B., and F.B.R. These challenges were displayed in a table under the headings “data preparation,” “analysis,” and “data interpretation.” Those specific to the OMOP CDM were further categorized using a generic harmonization process published by Henke et al., who proposed 9 steps to undertake harmonization of data to a common data model.^39^ The causes of challenges not related to the OMOP CDM were also described. The solutions used to enable federated analyses are described, along with recommendations for those performing federated analyses on critical care registry data.

## Results

### Participants and benchmarking

**Figure 1.**
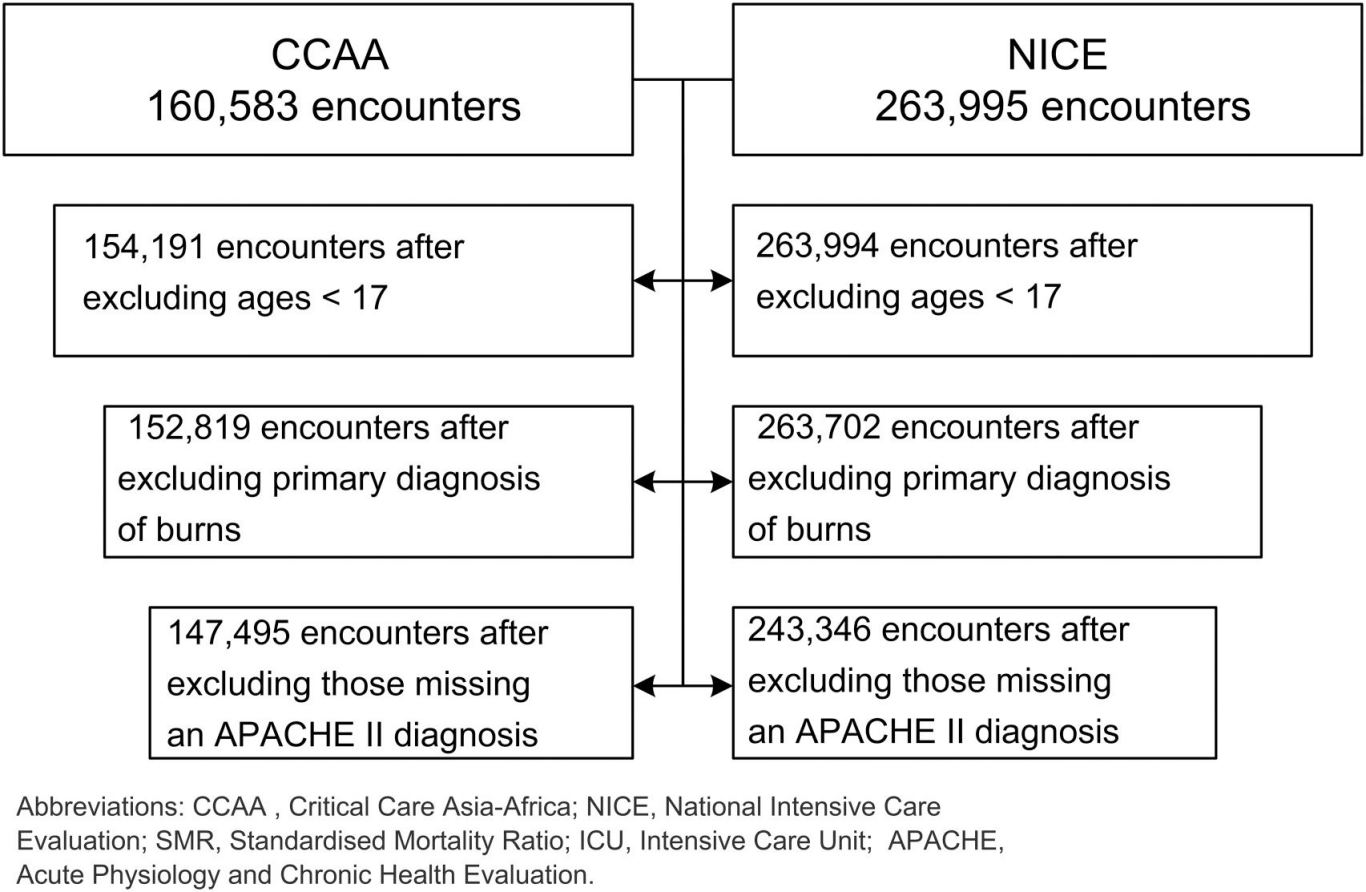
Flowchart of data inclusion and attrition. APACHE, Acute Physiology and Chronic Health Evaluation; CCAA, Critical Care Asia-Africa; ICU, Intensive Care Unit; NICE, National Intensive Care Evaluation; SMR, Standardised Mortality Ratio.

**Figure 2.**
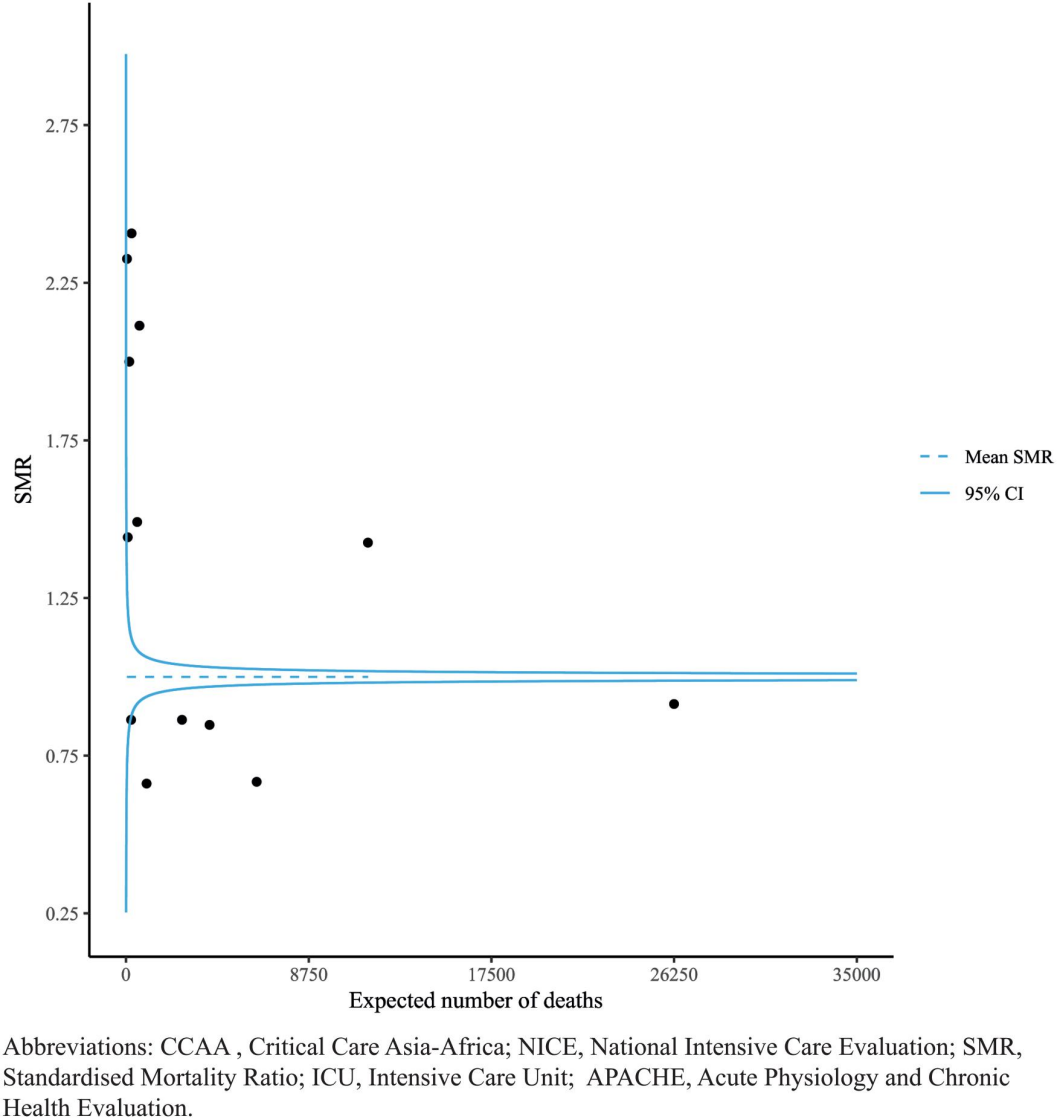
Funnel plot of APACHE II SMRs calculated over the period from July 2019 to December 2022. Each dot represents a registry (12 from CCAA and 1 from NICE). APACHE, Acute Physiology and Chronic Health Evaluation; CCAA, Critical Care Asia-Africa; ICU, Intensive Care Unit; NICE, National Intensive Care Evaluation; SMR, Standardised Mortality Ratio.

The data sources included 243 346 admissions in 74 ICUs from the NICE data source and 147 495 admissions in 186 ICUs from the CCAA data source, together representing 13 quality registries over the 3.5 years, see Table 1. Figure 1 describes the dataflow of the study. Availability and distribution of the APACHE II variables are described in Table S1 for CCAA and Table S2 for NICE. APACHE II SMRs ranged from 0.7 to 2.4 for all quality registries, see Figure 2. Analysis scripts were shared between the databases by which the federated analysis was successfully performed. The difference in OMOP CDM versions did not affect the variables used in this analysis, as the only difference between versions involved renaming variables which were not utilized in this study.

**Table 1.** Demographics and outcomes from the OMOP datasets.

Variable	CCAA	NICE
	*Demographics*	
Number of ICUs	186	74
Number of patients	147 495	243 346
Age Median (IQR)	55 (36-67)	66 (55-74)
Male, N (%)	82 996 (56.3)	151 755 (62.4)
	*Severity of illness*	
APACHE II score, Median (IQR)	14 (9.4-19.7)	15 (11.0-20.0)
	*Outcomes*	
ICU mortality, N (%)	30 636 (20.8)	25 424 (10.5)
ICU length of stay (Days), Median (IQR)	2.3 (1.2-4.7)	1.1 (0.8-3.0)

Abbreviations: APACHE, Acute Physiology and Chronic Health Evaluation; CCAA, Critical Care Asia-Africa; ICU, Intensive Care Unit; NICE, National Intensive Care Evaluation; OMOP, Observational Medical Outcomes Partnership.

### Challenges identified during the federated analysis

Seven challenges were identified during step 1, 4 of which related to data preparation, 2 to analysis plan, and 1 to data interpretation. Table 2 outlines these challenges, along with their impacts, the solutions implemented in step 2, and recommendations for future analyses. Of the 7 challenges, 4 related specifically to CDM transformation. These challenges arose from decisions made during the ETL process prior to this study regarding semantic mapping, structural mapping, and coverage of vocabularies.

**Table 2.** Challenges, their impacts, solutions, and recommendations for future analysis.

Challenge (Category)	Description	Impact	Solution	Recommendation
C1 (Data Preparation): Different SQL dialects between OMOP CDM databases	The 2 data sources are stored in different types of SQL Server (MS SQL & PostgreSQL) and therefore required different SQL dialects to query the server. This was an OMOP CDM related challenge that related to the ETL process in generic harmonization process.	Data from the 2 OMOP CDM databases could not be extracted using a shared SQL query.	The OHDSI SQLRender package was used to convert T-SQL code to PL/pgSQL.	Use the SQLRender package to translate queries where necessary. When using the package, ensure that queries are written in T-SQL, regardless of the servers used, as the package only translates T-SQL queries.
C2 (Data Preparation): Variation in base vocabularies used for concept codes	The 2 data sources occasionally use different base vocabularies to derive their concept codes within the OMOP CDM structure. This was an OMOP CDM related challenge that related to semantic mapping in the generic harmonization process.	Standardized concept codes could not be directly written into the shared data extraction script.	The data extraction script was adapted to build SQL queries based on a customizable configuration file in which the concept code for each variable was specified for each data source.	Proactively account for variations in concept codes by using a customizable configuration file for data extraction, rather than hardcoding concept codes into an SQL query.
C3 (Data Preparation): Variation in OMOP tables used to store concept codes	Specific concepts such as comorbidities and past medical history can be stored in multiple OMOP tables depending on interpretation. This was an OMOP CDM related challenge that related to structural mapping in the generic harmonization process.	Clinical concepts could not be directly linked to the OMOP tables in the SQL queries.	The data extraction queries were adapted to allow different tables to be specified in the customizable configuration file.	Anticipate that concept codes may be stored in different OMOP tables. Ensure the customizable configuration file and data extraction script can accommodate querying different tables based on user specifications.
C4 (Data Preparation): Lack of standardized vocabulary mappings for reasons for admission	The vocabularies used for reasons for admission did not have mappings to standardized OMOP vocabularies. This was an OMOP CDM related challenge that related to coverage analysis of vocabularies in the generic harmonization process.	The data extraction scripts for reasons for admission could not be shared between data sources.	The data extraction script was adapted so that reasons for admission were extracted separately for each data source.	Recognize when standardized extraction or federated analysis is not possible and use separate data extraction scripts per source. Standardize data formats as quickly as possible to resume federated analysis with shared scripts
C5 (Analysis plan): Lack of ICU identifiers in one data source	The 2 data sources applied different measures to enhance patient anonymity. Hospital identification was removed in NICE when loading data to the OMOP database. This challenge was a result of data storage decisions and was not related to the OMOP CDM.	SMR reporting at ICU level was not possible.	Data were aggregated to provide SMRs at the quality registry level.	Communicate data requirements between data sources before drafting the analysis plan. Confirm data availability early, using shared scripts if needed.
C6 (Analysis plan): Variation in vocabularies used to collect and store reason for ICU admission in the OMOP CDM	NICE stores reasons for admission as APACHE IV diagnosis codes. CCAA stores reasons for admission using SNOMED CT terms. This challenge was caused by variation in data collection methods and was not directly related to the OMOP CDM.	A shared analysis script could not be used to calculate APACHE II probability of mortality.	Separately from the federated analysis, an additional data source-specific mapping script was developed to map the reasons for admission to the APACHE II diagnostic codes. The APACHE II probability of mortality was then calculated using a shared script.	Recognize when standardized extraction or federated analysis is not possible and use separate data extraction or analysis scripts per source. Standardize data formats as quickly as possible to resume federated analysis with shared scripts.
C7 (Data interpretation): Different causes of missingness	The causes of missing data were different between the 2 data sources. This challenge was a result of variations in clinical practice, and resource availability between the NICE and CCAA sites, and was not related to the OMOP CDM.	The ability of APACHE II to predict mortality was impacted by degree and pattern of missingness.	The analysis was adapted to be context-sensitive regarding the meaning of missing data. In NICE data, missing data were handled as being within the normal range, as described in the APACHE II paper, while multiple imputation was used for CCAA data.	If data interpretation differs between sources, apply appropriate methods for each rather than enforcing a single approach.

Abbreviations: APACHE, Acute Physiology and Chronic Health Evaluation; C1-7, Challenge 1-7; CCAA, Critical Care Asia-Africa; ICU, Intensive Care Unit; NICE, National Intensive Care Evaluation; OHDSI, Observational Health Data Sciences and Informatics; OMOP CDM, Observational Medical Outcomes Partnership Common Data Model; PL/pgSQL, Procedural Language/PostgreSQL (used by PostgreSQL); SMR, Standardized Mortality Ratio; SQL, Structured Query Language; T-SQL, Transact-SQL (used by Microsoft SQL Server).

### Solutions implemented for each challenge

#### Technical issues (C1)

NICE and CCAA stored their data on separate servers, requiring different dialects for querying. NICE uses Transact-SQL (T-SQL) queries for their Microsoft SQL Server, while CCAA uses Procedural Language/PostgreSQL (PL/pgSQL) queries for their PostgreSQL server.^40^^,^^41^ However, OHDSI’s R package SQLRender only converts from T-SQL to other dialects and cannot perform the reverse conversion. Therefore, although author A.R. initially wrote the extraction query in PL/pgSQL, author D.P. manually rewrote the query to T-SQL. The analysis script then used SQLRender to automatically convert the query to PL/pgSQL.

#### Variations in standardized concept codes and tables (C2 and C3)

The OMOP Standardized Vocabularies allow the same clinical concept to be stored using different concept codes. This affected the physiology variables used for APACHE II. For example, leukocyte count could be stored as either “Leukocytes [#/volume] in Blood by Automated count” (3000905) or “Leukocytes [#/volume] in Blood by Manual count” (3003282). Thus, the data extraction analysis script was adapted to build SQL queries based on a customizable configuration file which specified a variable’s concept code. The concept codes could be based on either standard OMOP vocabularies or on custom codes stored in the data source only. The OMOP CDM allows concepts such as comorbidities and past medical history to be stored in various OMOP tables like Condition versus Observation, depending on interpretation. Therefore, the data extraction analysis script was adapted to allow different tables to be specified in the customizable configuration file.

#### Variations in collection and mapping of reasons for ICU admission (C4 and C6)

Calculation of the APACHE II mortality probability requires reasons for ICU admission to be coded as APACHE II diagnostic categories. Since 2018, NICE has derived these from the APACHE IV classifications. CCAA collects reasons for ICU admission as SNOMED CT codes, which are also mapped to APACHE IV classifications prior to mapping to APACHE II classifications. APACHE IV reasons for admission cannot be mapped to standardized OMOP vocabularies and were stored as unmapped data in the OMOP databases. Therefore, the data extraction script was adapted to extract reasons for admission separately for each data source. The data analysis script was also adapted to separately map reasons for admission from each data source into the APACHE II diagnostic categories. The calculation of the APACHE II probability of morality was then performed using a shared data analysis script.

#### Privacy concerns (C5)

The original intention described in the naive analysis plan was to report SMRs per ICU. However, NICE did not store identifying ICU information in their OMOP database due to privacy concerns and legal regulations with participating hospitals. Therefore, the analysis was adapted to report SMRs per quality registry instead of per ICU.

#### Data interpretation differences (C7)

Missing data were interpreted differently for each data source, due to differences in data collection context. For NICE, missing values were presumed to have been considered normal by clinicians and were thus handled as being within the normal clinical range for that specific variable, as described in the original APACHE II publication. This resulted in that component of the APACHE II score having a weight of “0,” indicating that the patient’s value for that variable did not contribute to an increased risk of death. For CCAA data, missing values could stem from resource constraints rather than clinician assumptions of normality. Therefore, multiple imputation with chained equations was used to impute missing values in the CCAA dataset.^33^^,^^42^ Multiple imputation for CCAA was performed using Predictive Mean Matching.^43–45^ Predictors were quality registry, ICU survival status, ICU length of stay, and the APACHE II component variables. Multiple imputation was performed 30 times, with 100 iterations per imputation, to produce 30 complete datasets in which missing values for each APACHE II component variable were imputed. The APACHE II probability of mortality was calculated separately per patient on each imputed dataset and the means over the imputed values were reported as median (IQR). For the SMRs, the expected number of deaths per quality registry was calculated separately on each imputed dataset. These were then combined using Rubin’s rules, and separate funnel plots were created using the point estimate and the upper and lower 95% confidence intervals, see Figure 2.^43^

## Discussion

This study demonstrates the feasibility of using quality registry data to perform a federated data analysis to benchmark clinical outcomes internationally. It describes the steps undertaken to perform federated analysis and explores the challenges associated with both the federated analysis and the interoperability of datasets following implementation of the OMOP CDM.

Challenges C2, C3, and C6 in Table 2 are directly linked to the flexibility of the OMOP CDM and its underlying terminology systems, which aim to harmonize data from diverse sources for research.^17^ OHDSI offers guidance, but when data do not exactly fit the model, OMOP CDM allows users flexibility in implementation. For example, OMOP CDM users are allowed to categorize comorbidities as observations, rather than conflating them with other medical conditions. The analysis script needs to accommodate these possible variations. As CDMs are increasingly used internationally, collaboration among researchers developing quality registries could enhance standardization in CDM implementation and the application of terminologies. Although achieving a common minimal dataset may be impractical, improving data findability and interpretability through the publication of data dictionaries and mapping frameworks could enhance future analyses.^46^ Guidelines such as the FAIRs metrics^46^^,^^47^ and initiatives like the LOGIC consortium^3^ already advocate for greater transparency in reporting data structures, publicly available analysis scripts, and metadata.

Pre-empting OMOP CDMs challenges of variation in implementation, OHDSI developed the web application “ATLAS,” enabling users to group concept codes into a single clinical entity.^48^ Our study group sought to use this tool in this analysis, but ATLAS currently supports only clinical characterization, population-level estimation, and patient-level prediction, not other study designs such as the benchmarking used in this analysis.^49^ Potential ATLAS users should be aware that installation requires a substantial level of technical expertise and possibly support from engineers familiar with the software. While collaboration between healthcare, research, and information technology sectors is growing, expertise in these areas remains limited, particularly in low and lower-middle-income healthcare research teams. This study group consisted of clinicians and data scientists, with some support from software engineers. This study reinforces the need for investment in these disciplines.

Challenge C1 was a consequence of OMOP CDM’s support for different types of SQL servers to host the database. The ODHSI community recognize that OMOP CDM’s utility is improved by its ability to support existing server structures. As many quality registries and EHRs are already operational, this flexibility allows curators to apply the CDM to existing datasets. However, this required development of analysis scripts to support different SQL dialects for federated analysis. While OHDSI offers a tool to translate queries from T-SQL dialect to other dialects, translation between dialects without using T-SQL is not supported. Researchers conducting federated analysis should be aware of these current limitations during analysis script development.

Missing data were handled differently for each data source, due to differences in presumed mechanisms by which the data were generated within the respective clinical contexts. For NICE, missing values for laboratory and physiological measures were presumed to have been considered normal by clinicians and were thus imputed using normal value imputation. For CCAA, it is understood that missing values may stem from resource constraints influencing access and use of tests, and therefore, multiple imputation was used. This challenge was not related to the OMOP CDM, although the variation in solutions highlights the necessity of considering data generation contexts and availability of metadata before developing the study protocol and analysis plan.

OHDSI recommends performing network studies by creating an analysis script using a single OMOP database and then running it on other datasets within the OHDSI data network.^50^ However, this study found this method, corresponding to the naive analysis initially attempted, to be infeasible, as there were discrepancies in data generation processes and availability among the data sources. An alternative approach, where authors first share a protocol and list of required metadata with collaborators, then edit the protocol, analysis plan, and analysis script based on metadata received, may mitigate some of these challenges. The “metadata” shared for each data source should include data dictionaries, concept mappings, results of OHDSI database characterization and data quality checks, and proportions of missingness for variables, along with any study or protocol specific information required. Other studies seeking to perform federated analysis emphasized the importance of assessing data availability, coverage, and quality before planning the analysis.^7^^,^^51^ The studies additionally recommended that collaborators validate OMOP concept code lists before analysis to mitigate the challenge of varied concepts codes.^52^

The use of real-world data for research is becoming increasingly important to gain insights into patient care and to gain knowledge related to the diagnosis, treatment, and prevention of diseases. Critical care services are increasingly at the forefront of response to disease, environmental, and political emergencies. Quality registry data have an important role in improving services, and standardization of quality registry data is making international analysis possible. This study is one of the first to use the OMOP CDM for federated analysis between international quality registries. Federated analysis can be a valuable tool for enabling research studies using existing accessible healthcare databases and can provide a mechanism for increasing interoperability. OMOP CDM can contribute to this under the condition that required metadata are discussed among network collaborators prior to the analysis. Future research should focus on how to identify challenges before development of the analysis, and how to mitigate them.

## Supplementary Material

ooaf052_Supplementary_Data

## Data Availability

Access to the data used in the analysis can be granted through the submission of an extraction form, which will be reviewed by a committee before access is granted. Extraction forms can be submitted here: NICE (website): https://www.stichting-nice.nl/extractieverzoeken.jsp CCAA (email): DAC@nicslk.com A list of R packages used in the analysis is available in Table S3. Data dictionaries, analysis scripts, and APACHE IV to APACHE II mapping tables are available on the figshare website for replication purposes. The Digital object identifier (DOI) for each item is listed below: Data dictionaries: https://doi.org/10.6084/m9.figshare.26268322.v1 Analysis scripts: https://doi.org/10.6084/m9.figshare.25837042.v2 https://github.com/aasiyahrashan/benchmarking-OMOP/releases/tag/v1.0.0 https://github.com/aasiyahrashan/SeverityScoresOMOP/releases/tag/v1.0 NICE mapping table: https://doi.org/10.6084/m9.figshare.25837876.v1 CCAA mapping table: https://doi.org/10.6084/m9.figshare.26268361.v1
