# Supplementary material for: Using the Observational Medical Outcomes Partnership Common Data Model for a multi-registry intensive care unit benchmarking federated analysis: lessons learned
Source: JAMIA Open. 2025 Jul 22;8(4):ooaf052. doi: 10.1093/jamiaopen/ooaf052 (PMC12282983; doi:10.1093/jamiaopen/ooaf052)
Supplement: ooaf052_Supplementary_Data [file ooaf052_supplementary_data.zip › Supplementary_table3-2[AU].docx]

**Supplementary table 3. List of R packages used in the analysis**

| **Package name** | **Link** |
| --- | --- |
| **conflicted** | https://cran.r-project.org/web/packages/conflicted/index.html |
| **data.table** | <https://cran.r-project.org/web/packages/data.table/index.html> |
| **DBI** | <https://cloud.r-project.org/web/packages/DBI/index.html> |
| **glue** | <https://cran.r-project.org/web/packages/glue/readme/README.html> |
| **googlesheets4** | <https://cran.r-project.org/web/packages/googlesheets4/index.html> |
| **lubridate** | <https://cran.r-project.org/web/packages/lubridate/index.html> |
| **mice** | <https://cran.r-project.org/web/packages/mice/index.html> |
| **odbc** | https://cran.r-project.org/web/packages/odbc/index.html |
| **openxlsx** | <https://cran.r-project.org/web/packages/openxlsx/index.html> |
| **purrr** | https://cran.r-project.org/web/packages/purrr/index.html |
| **readxl** | <https://cran.r-project.org/web/packages/readxl/index.html> |
| **RPostgres** | https://cran.r-project.org/web/packages/RPostgres/index.html |
| **SeverityScoresOMOP** | <https://github.com/aasiyahrashan/SeverityScoresOMOP/releases/tag/v1.0> |
| **SqlRender** | <https://github.com/OHDSI/SqlRender> |
| **TableOneDataFrame** | <https://github.com/aasiyahrashan/TableOneDatafram> |
| **tidyverse** | <https://cran.r-project.org/web/packages/tidyverse/index.html> |
