# Supplementary material for: Using the Observational Medical Outcomes Partnership Common Data Model for a multi-registry intensive care unit benchmarking federated analysis: lessons learned
Source: JAMIA Open. 2025 Jul 22;8(4):ooaf052. doi: 10.1093/jamiaopen/ooaf052 (PMC12282983; doi:10.1093/jamiaopen/ooaf052)
Supplement: ooaf052_Supplementary_Data [file ooaf052_supplementary_data.zip › Supplementary_table1.docx]

**Supplementary table 1. Availability and distribution of APACHE II component variables in the CCAA OMOP database**

| **Variable** | **Availability (%)** | **Distribution** |
| --- | --- | --- |
| Age, Median (IQR) | 100.0 | 55 (36.0 - 67.0) |
| Male, no. (%) | 100.0 | 82996 (56.3) |
| Organ failure/Immunocompromised, no. (%) | 100.0 | 8888 (6.0) |
| Renal failure, no. (%) | 100.0 | 4398 (3.0) |
| Emergency admission, no. (%) | 100.0 | 123310 (83.6) |
| Reason for ICU admission | 100.0 |  |
| Bicarbonate, Median (IQR), mmol/L | 53.5 | 21.9 (18.7 - 24.4) |
| Creatinine, Median (IQR), mg/dl | 73.1 | 1 (0.8 - 1.6) |
| Fraction of Inspired Oxygen, Median (IQR) | 75.0 | 0.4 (0.2 - 0.6) |
| Glasgow Coma Score, Median (IQR) | 96.7 | 15 (7.0 - 15.0) |
| Haematocrit, Median (IQR), % | 58.5 | 36 (30.0 - 41.0) |
| Heart rate, Median (IQR), beats/min | 98.1 | 94 (82.0 - 110.0) |
| Mean arterial pressure, Median (IQR), mmHg | 98.0 | 91 (80.0 - 102.7) |
| PaCO_2_, Median (IQR), mmHg^a^ |  |  |
| PaO_2_, Median (IQR), mmHg | 54.7 | 92 (67.7 - 130.0) |
| pH, Median (IQR) | 51.5 | 7.4 (7.3 - 7.4) |
| Potassium, Median (IQR), mmol/L | 70.7 | 4.1 (3.7 - 4.6) |
| Respiratory rate, Median (IQR), breaths/min | 98.1 | 21 (18.0 - 24.0) |
| Sodium, Median (IQR), mmol/L | 70.6 | 137 (133.0 - 141.0) |
| Temperature, Median (IQR), °C | 97.2 | 36.7 (36.7 - 37.0) |
| White cell count, Median (IQR), 10^9/L | 64.2 | 13.6 (9.0 - 24.0) |

^a^Not collected by CCAA. This is imputed as 40 mmHg for all patients when calculating the APACHE II score.

Abbreviations: IQR, interquartile range. BPM, beats per minute.

SI conversion factor: To convert creatinine to μmol/L, multiply values by 76.25.
