# Supplementary material for: Using the Observational Medical Outcomes Partnership Common Data Model for a multi-registry intensive care unit benchmarking federated analysis: lessons learned
Source: JAMIA Open. 2025 Jul 22;8(4):ooaf052. doi: 10.1093/jamiaopen/ooaf052 (PMC12282983; doi:10.1093/jamiaopen/ooaf052)
Supplement: ooaf052_Supplementary_Data [file ooaf052_supplementary_data.zip › Supplementary_table2.docx]

**Supplementary table 2. Database availability and APACHE II component variable distribution in the NICE OMOP database**

| **Variable** | **Availability (%)** | **Type** | **Distribution** |
| --- | --- | --- | --- |
| Age, Median (IQR) | 100.0 |  | 66 (55.0 - 74.0) |
| Male, no. (%) | 100.0 |  | 151754 (62.4) |
| Organ failure/Immunocompromised, no. (%) | 100.0 |  | 40586 (16.7) |
| Renal failure, no. (%) | 100.0 |  | 22996 (9.4) |
| Emergency admission, no. (%) | 100.0 |  | 26830 (11.0) |
| Reason for ICU admission | 100.0 |  |  |
| Bicarbonate, Median (IQR), mmol/L | 79.7 | Max. | 24.4 (22.0 - 27.0) |
|  |  | Min. | 22 (19.0 - 24.1) |
| Creatinine, Median (IQR), mg/dl | 89.7 | Max. | 0.9 (0.7 - 1.3) |
|  |  | Min. | 0.9 (0.7 - 1.2) |
| Fraction of Inspired Oxygen, Median (IQR) | 78.0 | Max. | 0.4 (0.2 - 0.6) |
|  |  | Min. | 0.3 (0.2 - 0.5) |
| Glasgow Coma Score, Median (IQR) | 98.7 | Max. | 15 (15.0 - 15.0) |
|  |  | Min. | 15 (14.0 - 15.0) |
| Haematocrit, Median (IQR), % | 91.0 | Max. | 37 (32.0 - 41.0) |
|  |  | Min. | 34 (29.0 - 38.0) |
| Heart rate, Median (IQR), beats/min | 98.1 | Max. | 98 (85.0 - 114.0) |
|  |  | Min. | 67 (58.0 - 79.0) |
| Mean arterial pressure, Median (IQR), mmHg | 97.3 | Max. | 101 (90.0 - 115.0) |
|  |  | Min. | 63 (56.0 - 71.0) |
| PaCO_2_, Median (IQR), mmHg | 76.7 | Max. | 40 (35.0 - 45.0) |
|  |  | Min. | 40 (35.0 - 45.0) |
| PaO_2_, Median (IQR), mmHg | 74.8 | Max. | 88 (74.0 - 113.0) |
|  |  | Min. | 81 (68.0 - 103.0) |
| pH, Median (IQR) | 76.5 | Max. | 7.4 (7.3.0 - 7.4.0) |
|  |  | Min. | 7.4 (7.3 - 7.4) |
| Potassium, Median (IQR), mmol/L | 89.1 | Max. | 4.4 (4.1 - 4.9) |
|  |  | Min. | 3.9 (3.6 - 4.3) |
| Respiratory rate, Median (IQR), breaths/min | 97.3 | Max. | 26 (22.0 - 31.0) |
|  |  | Min. | 12 (10.0 - 15.0) |
| Sodium, Median (IQR), mmol/L | 90.7 | Max. | 139 (137.0 - 141.0) |
|  |  | Min. | 136 (134.0 - 139.0) |
| Temperature, Median (IQR), °C | 95.2 | Max. | 37.4 (36.9 - 37.9) |
|  |  | Min. | 36.2 (35.7 - 36.7) |
| White cell count, Median (IQR), 10^9/L | 85.0 | Max. | 12.9 (9.6 - 17.2) |
|  |  | Min. | 11.1 (8.2 - 14.8) |

Abbreviations: IQR, interquartile range. BPM, beats per minute. Max, maximum value within 24 hours after ICU admission. Min, minimum value within 24 hours after ICU admission.

SI conversion factor: To convert creatinine to μmol/L, multiply values by 76.25.
